# Mechanistic modelling of allergen-induced airways disease in early life

**DOI:** 10.1038/s41598-024-83204-x

**Published:** 2025-01-02

**Authors:** Hannah J. Pybus, Prakrati Dangarh, Man Yin Melanie Ng, Clare M. Lloyd, Sejal Saglani, Reiko J. Tanaka

**Affiliations:** 1 Department of Bioengineering, Imperial College London, London, SW7 2AZ UK; 2 National Heart and Lung Institute, Imperial College London, London, SW7 2AZ UK; 3 Department of Respiratory Paediatrics, Royal Brompton Hospital, London, SW3 6NP UK

**Keywords:** Asthma, Pre-school wheeze, Allergen, In silico models, Mechanistic modelling, Computational models, Paediatric research, Computer modelling, Dynamical systems, Systems analysis, Asthma

## Abstract

Asthma affects approximately 300 million individuals worldwide and the onset predominantly arises in childhood. Children are exposed to multiple environmental irritants, such as viruses and allergens, that are common triggers for asthma onset, whilst their immune systems are developing in early life. Understanding the impact of allergen exposures on the developing immune system and resulting alterations in lung function in early life will help prevent the onset and progression of allergic asthma in children. In this study, we developed an in silico model describing the pulmonary immune response to a common allergen, house dust mite, to investigate its downstream impact on the pathophysiology of asthma, including airway eosinophilic inflammation, remodelling, and lung function. We hypothesised that altered epithelial function following allergen exposure determines the onset of airway remodelling and abnormal lung function, which are irreversible with current asthma therapies. We calibrated the in silico model using age appropriate in vivo data from neonatal and adult mice. We validated the in silico model using in vivo data from mice on the effects of current treatment strategies. The in silico model recapitulates experimental observations and provides an interpretable in silico tool to assess airway pathology and the underlying immune responses upon allergen exposure. The in silico model simulations predict the extent of bronchial epithelial barrier damage observed when allergen sensitisation occurs and demonstrate that epithelial barrier damage and impaired immune maturation are critical determinants of reduced lung function and asthma development. The in silico model demonstrates that both epithelial barrier repair and immune maturation are potential targets for therapeutic intervention to achieve successful asthma prevention.

## Introduction

Asthma affects approximately 10% of all children globally^1^, making it the most common chronic childhood disease. The pathophysiological features of asthma are chronic airway eosinophilic inflammation, airway remodelling, and airway hyperresponsiveness (AHR)^2^. AHR, a key marker of lung function, is defined as a predisposition of the airways to be narrowed excessively in response to an irritant that would cause little to no effect in non-asthmatic individuals. Irritants include allergens and a range of non-specific stimuli, e.g., cold air^3^. Exposure to allergens elicits an immune response, and continual exposure to allergens may lead to developing allergic asthma characterised by the pathophysiological features of asthma and allergic sensitisation (becoming sensitised to an innocuous agent with an exaggerated immune response upon re-exposure).

Frequent severe wheeze/asthma attacks in early childhood are the strongest risk factor for abnormal airway pathophysiology in early adulthood^4^. Up to half of all preschool children (1–5 years old) will suffer at least one episode of wheezing by their 6th birthday^5^. In most cases, wheezing episodes resolve by adolescence; however, for some preschool children aged between 1 and 5 years, this persists and progresses to asthma by school-age and adolescence through to adulthood. Since there is currently no cure for asthma once established, and the onset predominantly arises in childhood, it is vital to prevent wheeze onset and asthma development in preschool children.

Children are exposed to multiple environmental risk factors resulting in early life wheezing, including allergens and infections^6,7^, e.g., house dust mite (HDM) and respiratory syncytial virus (RSV). The airway epithelial barrier is the first line of defence against inhaled agents and may become dysfunctional when exposed to irritants such as allergens. The susceptibility of individuals to asthma development may be associated with epithelial barrier integrity and function, which may decrease with genetic mutations^8–11^. However, the effects of barrier function on wheeze onset and asthma development are difficult to assess in vivo. It is unethical to obtain repeated invasive lower airway samples from children to gain biological insights. Furthermore, assessing barrier damage in vitro is currently limited by relatively simple models only including up to 3 cell types, with limited ability to assess the impact of immune cells and other changes in the whole airway. Moreover, markers for barrier damage resolve quickly in vitro^12^. Therefore, in vivo and in vitro studies both have limitations, and investigating airway epithelial function in young children is particularly challenging.

Children are exposed to allergens whilst their immune system is still developing which may leave them more susceptible to developing asthma. The early life developmental phase of the immune system in children differs to adults and may be important in the onset of disease, i.e., age-dependent mechanisms (Th2 skewness) could lead to disease if they malfunction^13,14^. However, such age-dependent mechanisms are also difficult to assess in vivo.

To address these issues, in this study we developed an in silico model of the immune response to allergen exposure in early life by utilising existing age-appropriate in vivo mouse data; 4 protocols performed in neonatal mice^15,16^, and 1 protocol performed in adult mice^17^. We used the neonatal mouse data to construct our hypothesis and to inform the in silico model structure because the neonatal mouse models of HDM challenge are age-appropriate for allergen-induced pre-school wheeze and childhood asthma^15,16^. We used both the neonatal and adult mouse data to inform the in silico model parameter choices and provide insights into the expected dynamical trends. The in silico model represents an age-appropriate virtual mouse exposed to environmental agents. It serves as an in silico tool to test multiple environmental exposure scenarios during the developmental phase of the immune system, to interrogate the age-dependent mechanisms underlying the temporal dynamics of the immune response following environmental exposures, and identify possible interventions and how they may be effective.

Recently, we developed an in silico model of the immune response to RSV exposure in early life by integrating neonatal mouse data^18^. The simulations of the in silico model suggested that the accumulation of epithelial barrier damage can trigger type 2 immunity (high eosinophilic inflammation) resulting in impaired lung function. Here, we assume that similar underlying mechanisms may be important in the development of allergen-induced wheeze and asthma. We hypothesised that the age-dependent effects of immune maturation, alongside allergic sensitisation and sustained epithelial barrier damage, can explain a mechanism behind the progression of preschool wheeze to allergic asthma. We aimed to gain insights into the impact of slow immune maturation and allergen-induced epithelial barrier damage on the development of impaired lung function and how future interventions may be best suited to improve overall lung function. The objectives were to develop an interpretable in silico model that captures the development of allergen-induced wheeze and asthma, to investigate potential mechanisms underlying the development of allergen-induced wheeze and asthma that are difficult to assess clinically or experimentally, and focus specifically on the contribution of epithelial barrier repair and immune maturation.

## Results

### An in silico model of the immune response to HDM exposure

**Fig. 1:**
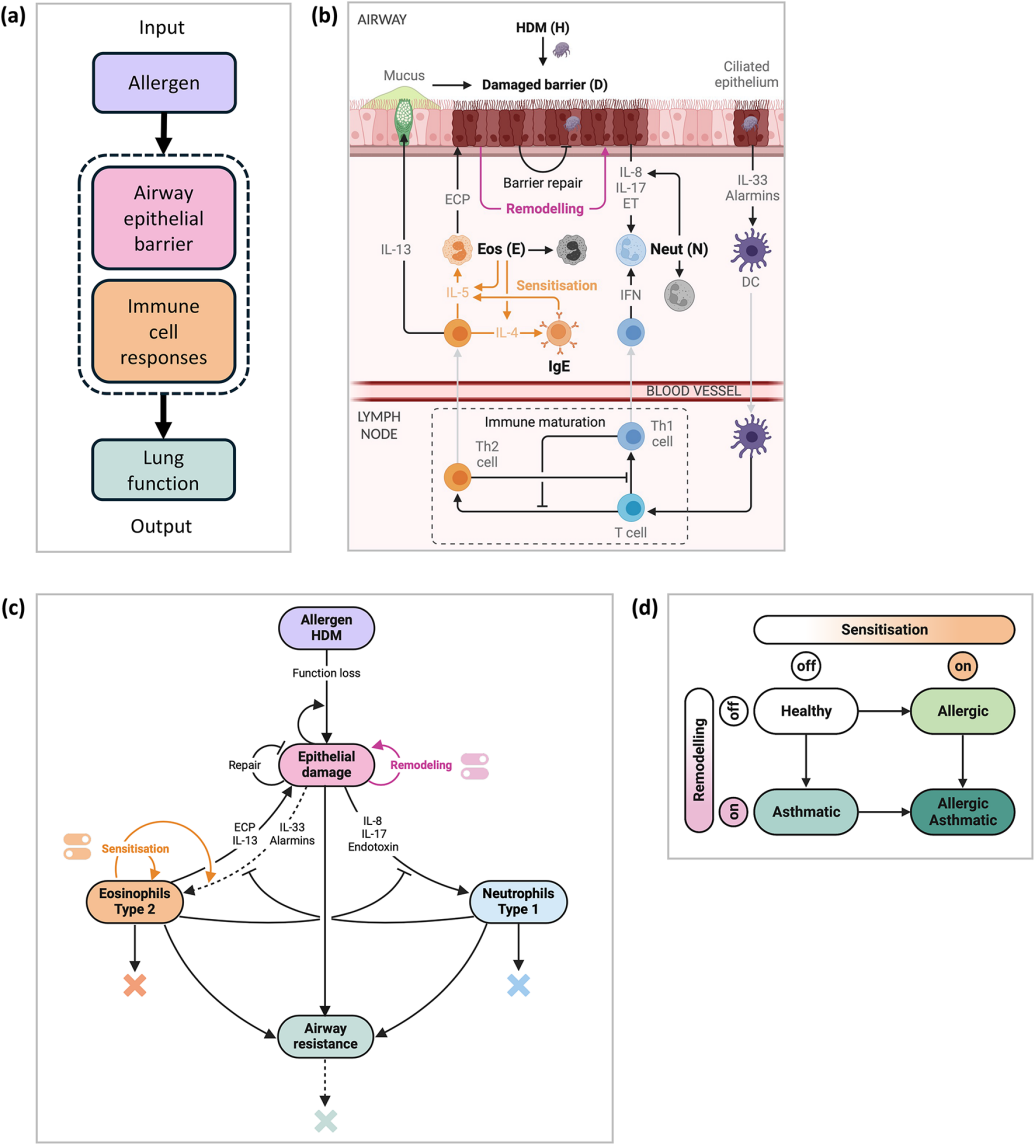
Development of an in silico model of the immune response to HDM exposure. (**a**) Overview highlighting the input, output, and key components of the in silico model. (**b**) Biological diagram of a cross section of the airway showing the key interactions and pathways included in the in silico model. Type 1 responses account for neutrophilic inflammation. Type 2 responses account for eosinophilic inflammation, allergic responses (IgE), and accompanying cytokines. The tissue-level airway resistance changes due to changes in the cell-level structure, which is caused by mucus accumulation, inflammation, and epithelial barrier damage. (**c**) Schematic of the in silico model with two switches (one for sensitisation and another for remodelling). Dashed lines indicate time-dependent rates. The colour coding for grouped cells follows that in Fig. 1b, where type 1 and type 2 responses are shown in blue and orange, respectively. (**d**) Long-term outcomes of the immune response described by 4 disease states (healthy, allergic, asthmatic and allergic asthmatic) defined by the on/off states of the two switches in the in silico model. Diagrams (**b**–**d**) created in BioRender (2024); (**b**) https://biorender.com/d54v750, (**c**) https://biorender.com/t10x595, (**d**) https://biorender.com/i93i862.

House dust mite (HDM) is a common aeroallergen that most children are exposed to in early life, which elicits an inflammatory immune response in susceptible individuals^19^. To investigate the underlying immune mechanisms associated with HDM-induced wheeze and asthma development, we developed an in silico model of the immune response to HDM in neonatal mice and the resulting airway resistance as a measure of lung function (Fig. 1a). As in a clinical setting, the in silico model assumes a significant increase in airway resistance is a sign of impaired lung function.

To investigate the impact of HDM-induced epithelial barrier damage on the development of impaired lung function, we considered the epithelial barrier damage as one of the main model states (Fig. 1a). The epithelial barrier is an active interface between the air we breathe and the underlying lung tissue. Healthy epithelial barriers are required for the maintenance of tissue homeostasis as they act as a defence to host tissue infections^10,20–22^. Epithelial barrier damage alongside type 2 immune cell responses forms an immunopathological unit that is thought to cause allergic airway inflammation^23^ (Fig. 1a).

The key interactions and pathways that are included in the in silico model of the immune response to HDM exposure were collated from literature and experimental observations (Fig. 1b). Breathing in HDM causes disruption, damage, and functional loss to the epithelial barrier, and HDM infiltrates the epithelial barrier and damages ciliated epithelial cells in the airway via protease activity^19^. The damaged epithelial cells secrete alarmins, such as interleukin (IL)-33, to initiate an immune response^15^ by recruiting innate lymphoid cells (ILCs) and activating dendritic cells (DCs). Activated DCs then migrate to the lymph node to initiate T cell differentiation allowing for type 1 (neutrophilic) and type 2 (eosinophilic) inflammatory responses. In early life, T cells preferentially differentiate to Th2 cells as opposed to Th1 cells, giving rise to Th2-skewing which reduces as the immune system matures. There is also evidence that type2 responses are more T-cell than ILC dependent in early-life^24^. Th1 and Th2 cells mutually inhibit through T-regulatory cells and the associated cytokines. Type 2 cytokines cause epithelial cell polarisation and disrupt the epithelial barrier function^25^. Three important type 2 cytokines secreted by Th2 cells are IL-5, IL-4, and IL-13. The in silico model captures the role of IL-5, IL-4, and IL-13 in the following processes: IL-5 recruits eosinophils to the site of damage^26^, where eosinophils secrete IL-5, further recruiting eosinophils^27^ and directly contributing to eosinophilic inflammation^28^; IL-4 increases IgE that increases IL-5 which recruits more eosinophils that also secrete IL-4, thus creating a positive feedback loop of IL-4 and IL-5 production via IgE and eosinophils during sensitisation (orange arrows in Fig. 1b); IL-13 contributes to the overproduction of mucus by activating the mucus producing gene in goblet cells, Muc5ac^24,29,30^. Eosinophils also release large granules and toxins, such as eosinophilic cationic protein (ECP), to degrade foreign entities^31^; however, ECP causes further damage to the epithelial barrier^32^. Further damage of the epithelial barrier is also caused by toxins secreted by microbes^33^ whose growth and infection are enhanced by accumulation of mucus^33^ although the primary role of the mucus is to remove respiratory irritants.

To develop an interpretable in silico model of the immune response to neonatal HDM exposure, we reduced the biological diagram (Fig. 1b) to a simplified schematic (Fig. 1c) that retains the key features of the immune response to HDM. The simplified schematic (Fig. 1c) describes net effects of all the pathways shown in the biological diagram (Fig. 1b). The in silico model is formulated by a system of four ordinary differential equations (ODEs) that describe the rate of change in the extent of epithelial barrier damage, eosinophilic inflammation, neutrophilic inflammation, and airway resistance (where increased resistance from baseline suggests wheeze/asthma) in response to HDM exposure (see Methods). The epithelial barrier damage includes both physical and functional loss, and eosinophilic inflammation is used as a representative marker to group all type 2 responses, whilst the neutrophilic inflammation is a marker of all type 1 responses. AHR describes the overall changes in the lung function seen in asthma and comprises different components, including increased airway resistance, which may result from inflammation, structural or functional changes in the barrier^34–36^. The in silico model also accounts for both lung growth and maturity of the immune system in early life, where lung growth is indicated by the change in the airway resistance resulting from increased airway size and maturation of the immune system by the change in the Th2 skewing (dotted lines Fig. 1c).

The in silico model includes two switches, one for allergic sensitisation and another one for airway remodelling, whose states are assumed to represent disease states where symptoms of asthma get worse as switches turn on (Fig. 1d). A healthy lung state refers to no sensitisation or remodelling, an allergic state includes sensitisation, an asthmatic state includes remodelling, and finally, an allergic asthmatic state includes both sensitisation and remodelling. This assumption is based on current literature and experimental observations in neonatal mice^16^ where changes in both the immune system and airway structure are required to make a lasting impact on the airway resistance, and hence, lung function. The irreversible changes of sensitisation and remodelling that impact lung function occur by week 4 in neonates because there is a significant increase in allergen-specific IgE by week 4, indicating allergic sensitisation^16^, and subepithelial reticulin thickness reaches a maximum by week 4 with little to no resolution post removal of HDM challenge, suggesting airway remodelling^16^.

We assume that the sensitisation switch in the in silico model turns on once the extent of eosinophilic inflammation (representative marker of type 2 responses) surpasses a threshold including HDM-specific IgE levels above a certain value. The remodelling switch in the in silico model is assumed to turn on once the extent of barrier damage surpasses a threshold. It assumes minor insults to the barrier are repaired through epithelial replenishment, whilst larger insults trigger an aberrant/dysregulated repair process. For example, laying down matrix structures in the epithelium instead of functional epithelial cells leads to the loss of barrier functionality making it more damaged/defective.

**Fig. 2:**
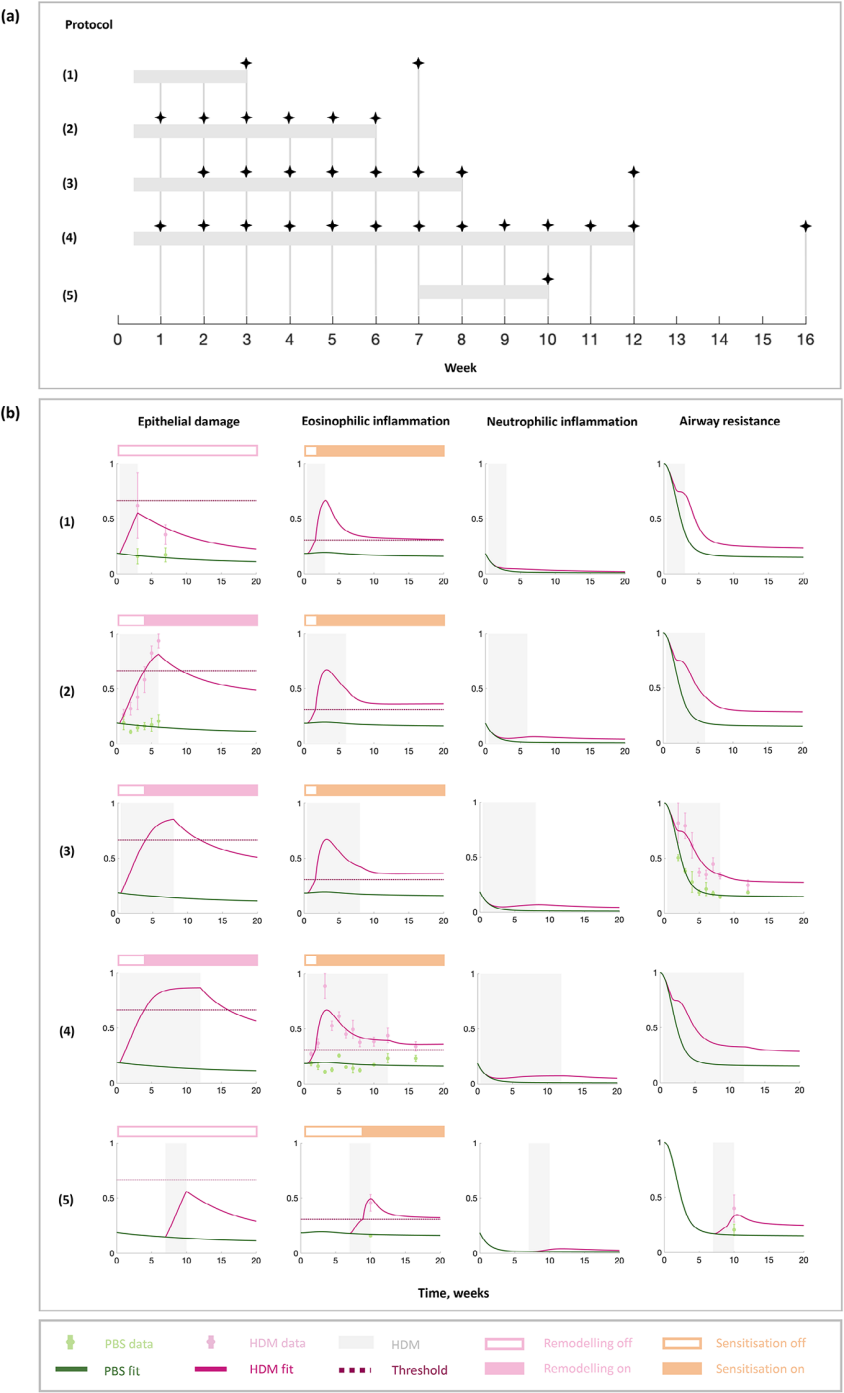
In silico model fitting to experimental data. (**a**) Experimental protocols of published in vivo mouse model studies we used for model fitting. Shaded regions indicate HDM challenge present and stars represent the timings of experimental data measurements (weeks from birth). (**b**) Model simulations of the parameterised in silico model for each of the experimental protocols. The data obtained for each protocol are (**1–2**) epithelial barrier damage (IL-33)^15^, (**3**) airway resistance^16^, and (**4**) eosinophil cell counts^16^ from a neonatal mouse model, and (**5**) eosinophil cell counts and airway resistance^17^ from an adult mouse model. Shaded regions indicate HDM challenge present and the dots with error bars indicate the mean data measurements with SEM. The green lines indicate the fit to PBS data and the dark pink lines the fit to HDM data. The dotted horizontal lines in the epithelial damage and eosinophilic inflammation plots indicate the thresholds for the remodelling and sensitisation switches, respectively. The resulting switch states are shown by the colours of the bars above the plots, with the on-states represented by pink and orange for remodelling and sensitisation switches and the off-states represented by white.

The model parameters were obtained by parameter optimisation using a Genetic Algorithm (GA) for experimental data from multiple experimental protocols (Fig. 2a) on both age-appropriate neonatal mice and adult mice on the epithelial barrier damage^15^, eosinophilic inflammation^16,17^, and airway resistance^16,17^ (Fig. 2b) as a measure of lung function. Our parameterised in silico model replicates the qualitative features of the experimental observations (Fig. 2b)^15,16^: epithelial damage (IL-33) steadily increases with HDM challenge; eosinophilic inflammation peaks at week 3 and remains elevated throughout but reduces after week 3 and further reduces once HDM challenges subside; the airway resistance, although greater than control, appears to decline throughout the experiment due to the growth and development of the mice. HDM causes an increase in airway resistance from baseline; a significant increase in airway resistance in response to HDM suggests AHR.

### In silico model simulations of current treatment strategies

**Fig. 3:**
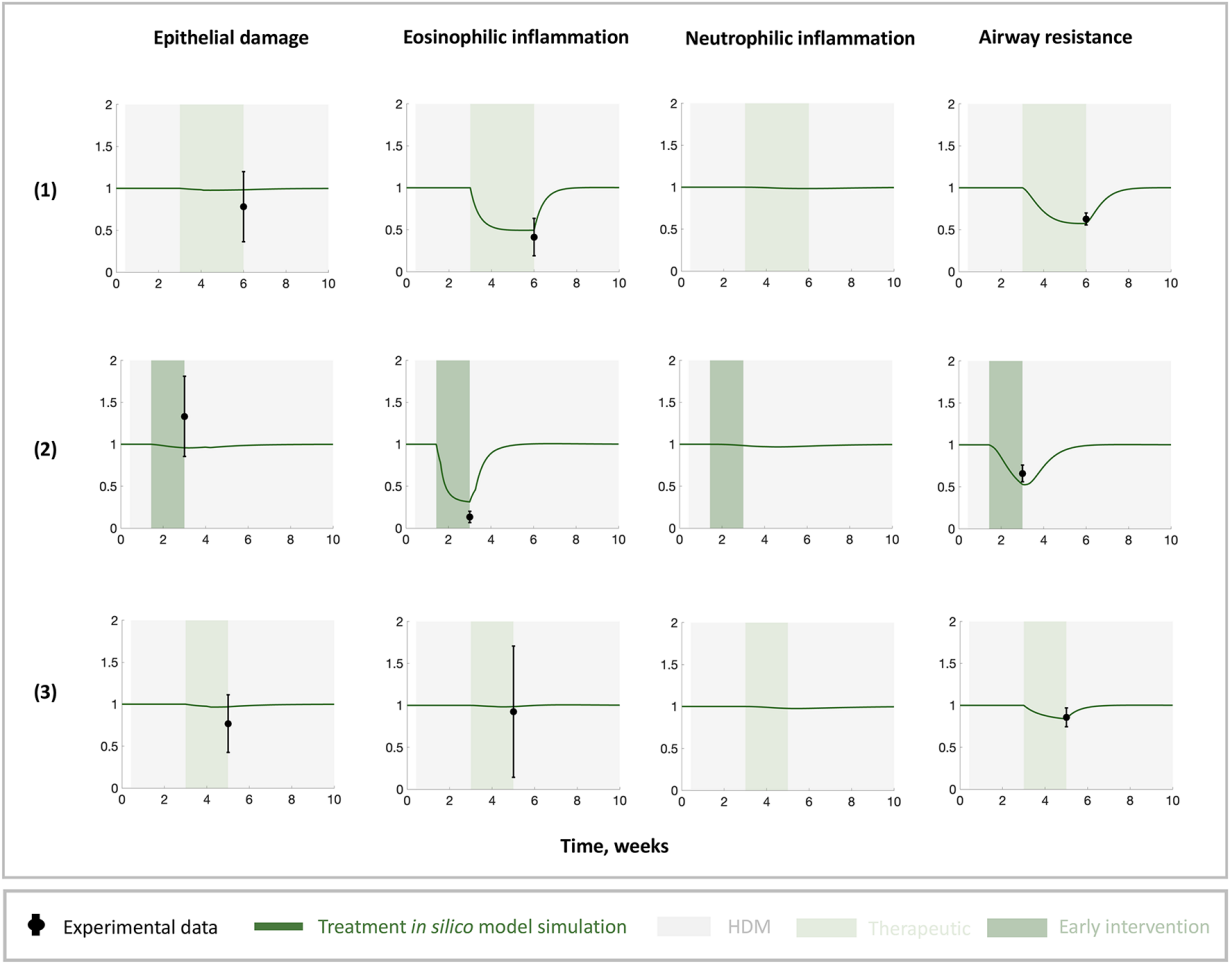
Validation of the in silico model against current treatment strategies. (**1**) Corticosteroid applied at week 3, *i.e.*, after the disease onset^15^, (**b**) corticosteroid applied at day 10, *i.e.*, before the disease onset^37^, and (**c**) anti-IL-13 use at week 3, *i.e.*, after the disease onset, in a mouse model^15^. All data points (black circles with SEM error bars) and simulation dynamics (solid green lines) are plotted as a fold change in treatment application vs placebo with HDM present from day 3 of life. Grey shaded regions indicate HDM present, dark green shaded regions indicate treatment interventions before disease onset, light green shaded regions indicate therapeutic treatment use after disease onset.

We confirmed that the parameterised in silico model can capture the behaviour of in vivo experiments to which the model has not been fitted to so that the model can be used to make predications for other in vivo experiments. We confirmed that the in silico model simulations can capture the main features of the experimental observations reported in published studies for three treatment strategies using corticosteroids and one monoclonal antibody given either before or after the onset of disease (Fig. 3). Corticosteroids are modelled to partially block their targeted pathways (by a half) since they are inhaled, whilst biologics are modelled to entirely block their targeted pathways since they are administered systemically. The in silico model simulations agree with the published studies showing that: there is a reduction in eosinophilic inflammation and airway resistance with no change in epithelial damage following corticosteroid treatment^15^; inflammation and airway resistance resolve by the end of the treatment if corticosteroid is used as an early intervention^37^; there is a small reduction in airway resistance with no change in damage following anti-IL-13 treatment^15^. Our simulation results suggest that current treatment strategies are not disease modifying when introduced after disease onset or if treatment ends whilst HDM exposure persists (Fig. 3, weeks following treatment use). This is shown by the fold change dynamics returning to 1 after the treatment ends, indicating that there is no difference between the treated and untreated cases. A similar observation held true even when we changed the timing and the duration of treatment application (Figure S1), highlighting the need to investigate potential targets for disease modification and how they may be effective in improving long term lung function.

### Time of HDM exposure impacts long term lung function

Before investigating potential targets for disease modification, we need to set out how we can evaluate the effects of simulated treatments. One way is to look at the simulated time-course dynamics for a single scenario, another is to do a systematic assessment of different timings and durations of treatment and allergen exposure. It is known that both allergen sensitisation and airway remodelling occur in the development of allergic asthma^16^; however, the impact of the timing of HDM exposure on long-term lung function is not clear. It is unethical to test a large number of different protocols using mouse models but is simple to do using in silico models. Therefore, we utilised our validated in silico model, representing a virtual mouse, to test different HDM protocols.

The parameterised in silico model represents the mean immune response to HDM of a nominal virtual mouse. We also obtained an alternative virtual mouse, which has different threshold values of the switches from the nominal values, resulting in switches being turned on in a different order, yet retains the qualitative features of the immune response to HDM (Figure S2).

**Fig. 4:**
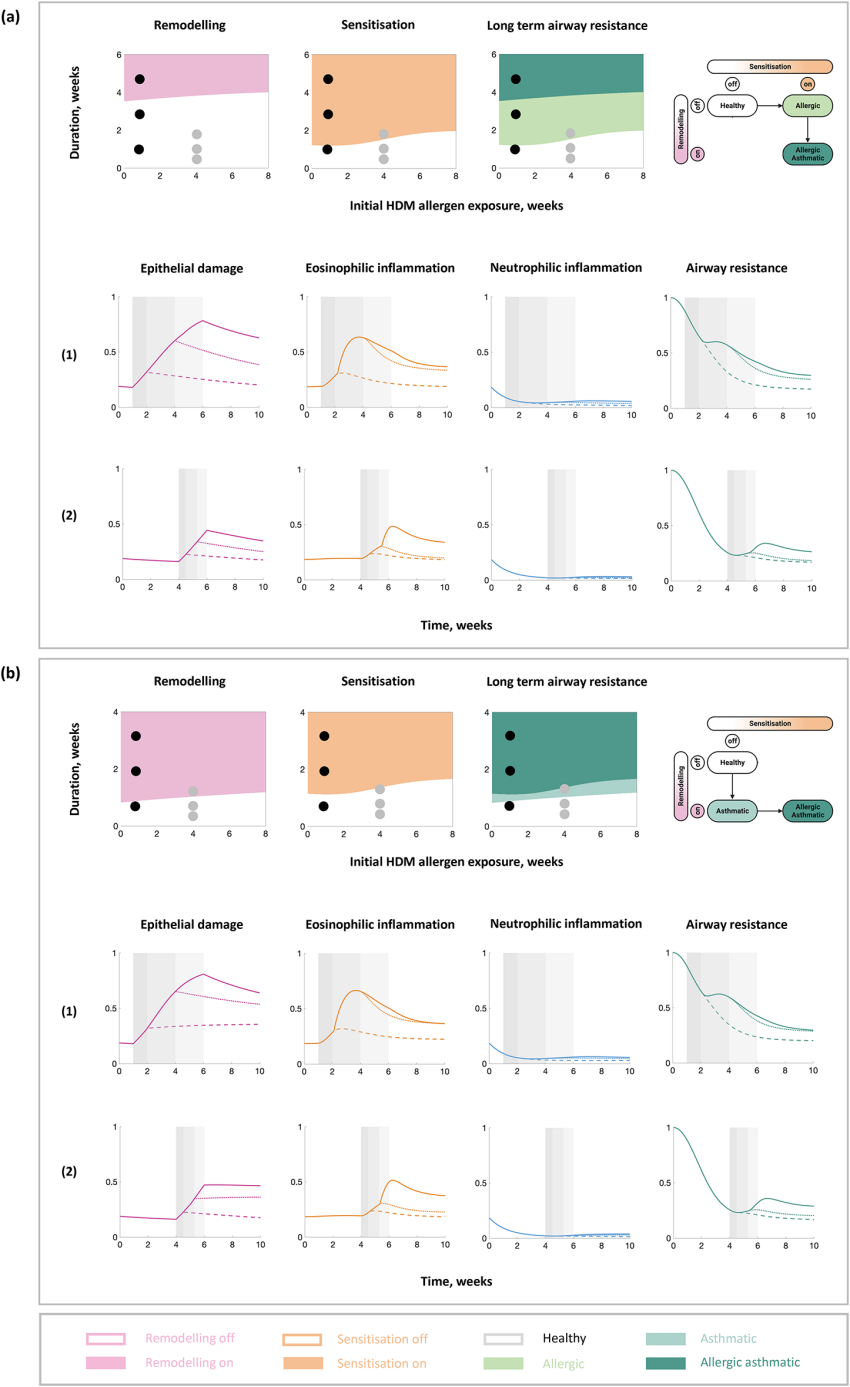
In silico long-term outcomes of HDM exposures of varied initial times and durations. Results for (**a**) the validated in silico model of the nominal virtual mouse and (**b**) the in silico model with modified parameters (an alternative virtual mouse, Figure S2). (Top) Switch plots that summarise the simulation results of long-term disease states as a result of HDM exposure for different durations starting at different timings. (Middle and bottom) Example dynamics as a result of the in silico model simulation for three representative scenarios of HDM exposure (**1**) at week 1 for 1 (dashed lines), 3 (dotted lines), and 5 (solid lines) weeks and (**2**) at week 4 for 0.5 (dashed lines), 1.3 (dotted lines), and 2 (solid lines) weeks. The black dots in the top rows of switch plots indicate the three representative scenarios in (**1**) and the grey dots in the top rows of switch plots indicate the three representative scenarios in (**2**).

To investigate the long-term outcome in HDM sensitisation (indicated by high allergen-specific IgE, i.e., type 2 cells), airway remodelling, and airway resistance (as a measure of lung function), we simulated early life immune response to HDM for different durations and initial exposures in a nominal and an alternative virtual mouse (Fig. 4). The long-term outcomes in lung function differ for the nominal and alternative virtual mice (Fig. 4a,b, respectively) due to the change in switch order. The switch plots (Fig. 4, top row) show how the duration and the timing of the initial exposure of HDM affects immune response dynamics. For example, introducing HDM a week after birth of a virtual mouse and removing it a week later leads to a small perturbation in the dynamics of the epithelial barrier damage, eosinophilic inflammation, and airway resistance that returns to a healthy state (Fig. 4, dashed lines). Removing HDM later (three weeks after introduction in the in silico model, that is, week four in a mouse) leads to a larger perturbation and may lead to sensitisation or remodelling (Fig. 4, dotted lines). Removing HDM much later (five weeks after introduction in the in silico model, that is, week six in a mouse) leads to a much larger perturbation in the dynamics and to changes in the airways with both sensitisation and remodelling (Fig. 4, solid lines).

The in silico model results also suggest that delaying the initial allergen exposure is beneficial to the long-term lung function since a longer exposure of allergen is required to create a diseased effect, i.e., both the sensitisation and remodelling switch remain off for a longer duration of time if the initial exposure of HDM is in later life compared to early life (Fig. 4). In contrast, earlier and longer exposures to HDM worsens symptoms (as both sensitisation and remodelling occur) and the resulting long-term lung function (Fig. 4, increased size of dark green regions of switch plots as the initial exposure time decreases). Reducing the amount of time spent with allergens in the environment aligns with efforts to prevent asthma; however, allergen avoidance alone has not been successful^38^ and in some cases, it is not possible to avoid allergens. Therefore, alternative strategies and potential targets need to be investigated.

### Impaired airway epithelial barrier repair impacts long term lung function

Epithelial barrier repair includes both the restoration of the physical barrier and the immune function of the barrier. Our previous work^18^ suggests epithelial barrier repair plays an important role in the development of disease, and therefore should be considered as a potential treatment target. Assessing the rate of epithelial barrier repair in vivo or modifying it in vitro are either too invasive or not possible. However, it is simple to modify the rate of barrier repair and assess the predicted outcomes in lung function using an in silico model. Therefore, we utilised the validated in silico model of the immune response to HDM to test the effects of varied rates of epithelial barrier repair.

**Fig. 5:**
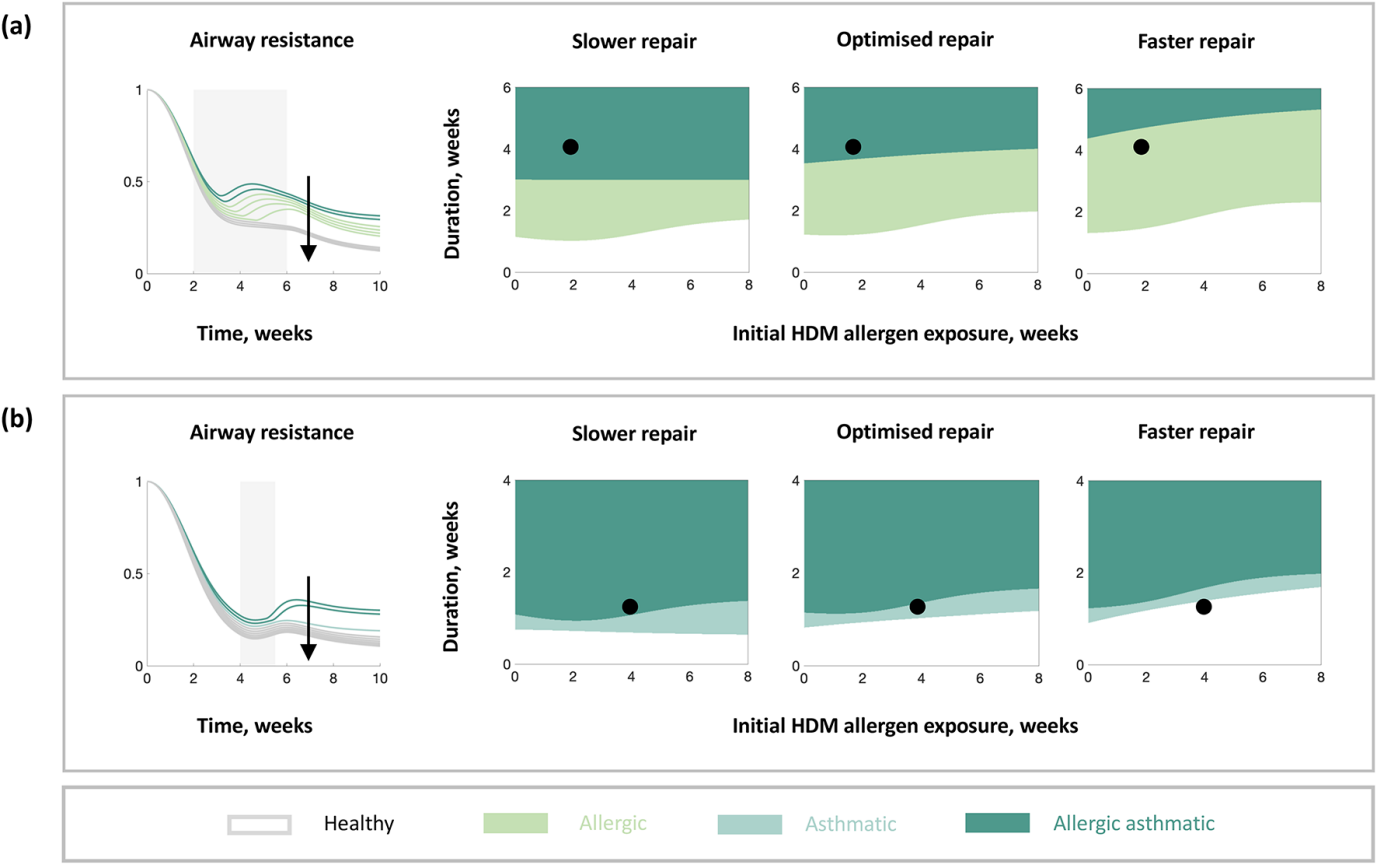
In silico model simulations of airway resistance for different rates of epithelial barrier repair. Dynamical responses (left) and long-term outcomes (right) in airway resistance for different initial times and durations of HDM exposure, and rate of epithelial barrier repair (faster in the direction of the arrow). Results for (**a**) the validated in silico model of the nominal virtual mouse and (**b**) the in silico model with modified parameters (an alternative virtual mouse, Figure S2). The grey shaded region indicates when HDM is present, with the corresponding initial HDM exposure and the duration shown as the black dot on the switch plots. The line colours in the dynamical plots correspond to the switch state regions in the switch plots, except the grey lines which correspond to the white region on the switch plots (for visibility). Slower repair refers to a reduced epithelial barrier repair rate (0.5x), optimised repair refers to the optimised epithelial barrier repair rate (1x), and faster repair refers to an increased epithelial barrier repair rate (1.5x).

We evaluated the in silico dynamics of airway resistance for different durations and initial exposures of HDM for virtual mice with different rates of epithelial barrier repair (Fig. 5). For the same duration and initial exposure of HDM (Fig. 5, black dots), there is a change in the long-term disease states when the rate of epithelial barrier repair is varied. The simulated airway resistance is decreased if the epithelial barrier repair is faster (Fig. 5, black arrows). Decreased airway resistance mimics improvement in the long-term lung function, whilst increased airway resistance suggests changes in the airway such as allergic sensitisation and airway remodelling, leading to a long-term reduction in lung function. The timings of HDM exposure that result in remodelling in the in silico model change depending on the rate of epithelial barrier repair, i.e., the combinations of durations and initial exposures that cause a change from light to dark green (Fig. 5a) or white to light green (Fig. 5b), change depending on the rate of epithelial barrier repair. However, the timings of HDM exposure that result in allergic sensitisation in the in silico model are unaffected by changes in the epithelial barrier repair. Taken together, the in silico model results suggest that faster rates of epithelial barrier repair could be beneficial in preventing asthma progression.

### Slow immune maturation leaves host susceptible

**Fig. 6:**
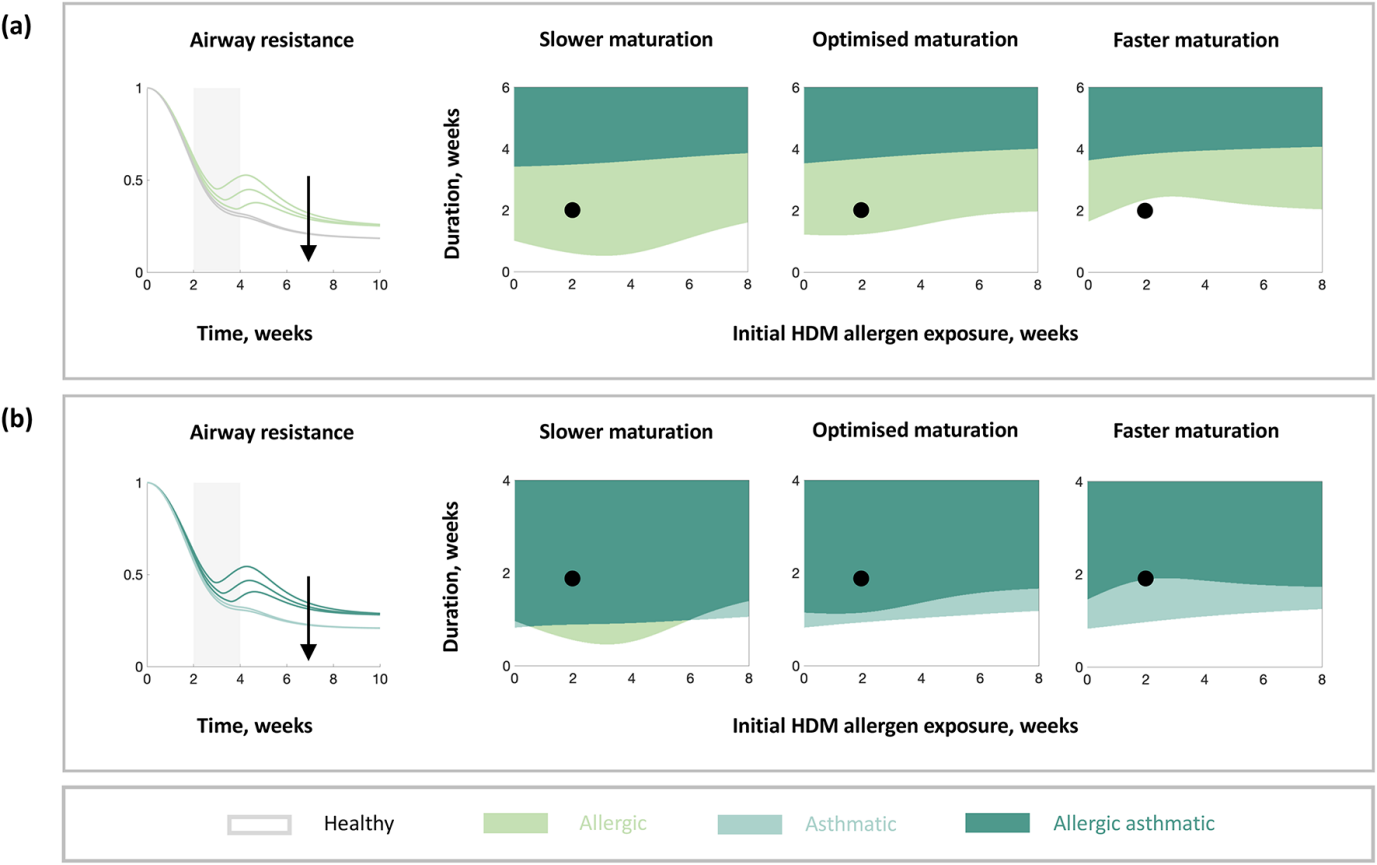
In silico model simulations of airway resistance for different rates of immune maturation. Dynamical responses (left) and long-term outcomes (right) in airway resistance for different initial times and durations of HDM exposure, and rate of immune maturation (increases in the direction of the black arrows). Results for (**a**) the validated in silico model of the nominal virtual mouse and (**b**) the in silico model with modified parameters (an alternative virtual mouse, Figure S2). The grey shaded region indicates when HDM is present, with the corresponding initial HDM exposure and the duration shown as the black dot on the switch plots. The line colours in the dynamical plots correspond to the switch state regions in the switch plots, except the grey lines which correspond to the white region on the switch plots (for visibility). Impaired refers to a reduced immune maturation rate (0.75x), optimised refers to the optimised immune maturation rate (1x), and improved refers to an increased immune maturation rate (1.25x).

The early life developmental phase affecting the maturation of the immune system is an important distinguishing feature between children and adults and should therefore be investigated as a potential treatment target. To investigate the impact of developmental changes on long-term lung function, we evaluated the simulated airway resistance for virtual mice with different rates of immune maturation for different durations and initial exposures of HDM (Fig. 6). There is little to no change in eosinophilic inflammation and airway resistance when varying lung growth (Figure S3). Different rates of immune maturation resulted in a change in the long-term disease states even for the same duration and initial exposure of HDM (Fig. 6, black dots). Slower rates of immune maturation, i.e., slow rates of change in Th2 skewing, increased airway resistance, suggesting that they are detrimental to lung function (Fig. 6). Therefore, slow rates of immune maturation potentially leave children susceptible to asthma development, e.g., premature babies have a higher risk of asthma development than full term babies. Faster rates of immune maturation are beneficial to lung function (Fig. 6).

The timings of HDM exposure that result in allergic sensitisation in the in silico model change depending on the rate of immune maturation; however, the timings of HDM exposure that result in remodelling in the in silico model are unaffected by changes in immune maturation. Slower rates of immune maturation increase the eosinophilic inflammatory response to HDM in early life, whilst faster rates of immune maturation decrease the eosinophilic inflammatory response to HDM in early life (Figure S3). Furthermore, the time taken for remodelling and sensitisation to occur increases as the rate of immune maturation increases; for faster rates of immune maturation, sensitisation may not occur (Figure S3). Taken together, the in silico model results suggest that increasing the rate of immune maturation could be beneficial in preventing asthma progression.

## Discussion

In this study, we developed an in silico model of the immune response to HDM exposure in early life to investigate the downstream impact of allergen exposure on lung function. The in silico model represents the key interactions of the immune response, identified based on literature and experimental evidence, and is parameterised using the experimental data from both neonatal and adult mice. Crucially, the in silico model utilises data from age-appropriate mouse studies to inform the model construction and parameterisation. The in silico model assumes two switches to account for the irreversible change in the airways due to allergic sensitisation and airway remodelling and includes time-dependent exponential functions to account for lung growth and immune maturation during the development phase in early life. We validated the in silico model using in vivo data from mouse models that assessed the effects of several treatment strategies that are currently in use in clinics. Thereafter, we used the validated in silico model to simulate different HDM exposure protocols to test multiple environmental exposure scenarios, interrogate the mechanisms underlying the temporal dynamics of the immune response following environmental exposures, and identify possible intervention targets.

One of the key findings from the in silico model simulations in this study is that sustained epithelial barrier damage and allergic sensitisation, upon HDM exposure, can capture the development of airway pathology. It is in line with the finding from our previous computational modelling study which suggests that a compromised epithelial barrier repair capacity can trigger type 2 immunity (high eosinophilia) upon RSV exposure in early life^18^. These results highlight the potential important role of the epithelial barrier in the development of airway pathology. Another key finding from the in silico model simulations is that the rate of immune maturation from neonates to adult mice also plays an important role in the development of airway pathology. The in silico model results for different timings of HDM exposure show that changes in the barrier repair rate cause changes in the timing when remodelling may occur, whilst changes in the immune maturation rate cause changes in the timing when allergic sensitisation may occur. This suggests that both the epithelial barrier repair and immune maturation need to be targeted together for potential disease modification. These results are robust to parameter variations which may describe a variety of different phenotypes (Figure S6).

It should be noted that Th2 skewing begins to plateau as the immune system matures (around week 6 in mice), and as a result, treatments that modify the rate of immune maturation may only be effective in early life before irreversible changes in the airways occur. Therefore, continuing treatment targeting immune maturation past early life (as a therapeutic) may have no long-term benefit. As a means of early intervention, however, agents modifying the rate of immune maturation may be promising in asthma prevention.

The in silico model allowed us to investigate mechanisms that are difficult to assess experimentally or clinically. This work upholds the 3Rs principles (replacement, reduction, refinement) by developing computational models utilising existing mouse data without conducting any new animal experiments. Using the in silico model developed and validated in this study, we can simulate varying HDM protocols, i.e., introduce allergen and remove allergen at varying timings, to assess the ensued dynamical changes in epithelial barrier damage, eosinophilic inflammation, and lung function. In the future, the in silico model can be used to evaluate the effects of a generalised aeroallergen/irritant that children may be exposed to in early life without the need for additional animal models. It will require concerted efforts within respiratory medicine and computational modelling communities to generate a database of existing in vivo mouse data that can be used to fit to computational models because many animal models of allergic airways disease exist but with significantly diverse protocols. Creating such a database will also provide insights into which areas of data collection are lacking.

To date, multiple in silico methods have been used to model the airways and asthma development. Statistical and data-driven methodologies have been used to group children by pathophysiological features^39^ and distinguish phenotypes^5^. Other statistical studies have used longitudinal data clustering to derive exacerbation trajectories from childhood through to adolescence^40^. Since current statistical methods do not interrogate the potential mechanisms underpinning asthma development, more interpretable mechanistic modelling approaches are required alongside. One relevant mechanistic in silico model of asthma describes the interaction between cytokine activation and airway remodelling, showing that repeated exacerbations/exposures to irritants can cause irreversible damage and changes in the airway composition^41^. Other in silico approaches investigate: the relationship between immune responses and the subsequent alveolar tissue damage in allergen-induced asthma^42^; the macrophage response to RSV in normal and asthmatic conditions^43^; the inflammation resolution speed in asthmatic airways^44^; the regulation of Th1 and Th2 cells in asthma development^45^; the properties of the mechanical microenvironment^46,47^ and contractile force generation in the airways^48^ contributing to pathophysiology. Other relevant models outside the context of asthma include those investigating the interaction between infiltrated pathogens, immune response, and skin epithelial barrier integrity in the onset of atopic dermatitis (AD)^49^, since AD exacerbations share similar factors with exacerbations of pre-school wheeze and asthma. Despite there being multiple modelling approaches for asthma development, most are based on findings in adults, which are thought to have a different mechanism to early life^16^. Little is known about the mechanisms underpinning early-life allergen-induced wheeze and asthma.

To our knowledge, this is the first in silico mechanistic model of the immune response to HDM exposure in early life calibrated with age-appropriate data. Our work helps close the gap in what is known in children and adults and provide an interpretable in silico tool that can be used to assess airway pathology and the underlying immune response mechanisms upon HDM exposure. The work presented forms a basis for modelling the effects of multiple environmental exposures on long-term lung function in the future.

To start to bridge the gap from mouse to human studies, we validated the in silico model against clinical data from children that assessed the effects of several treatment strategies, such as anti-IL5 antibody, currently in use in clinics (Figure S4). In agreement with clinical observations, the in silico model shows that there is a reduction in inflammation with no change in epithelial damage following Mepolizumab treatment^50^, and there is an improvement in lung function following anti-IL-4r antibody (Dupilumab) treatment^51^. While the in silico model developed in this study has potential to be used to simulate the effects of allergen exposure in children, further calibration using data from children will help to advance the in silico model to simulate different environmental exposures and treatment regimes. This would provide an in silico tool to predict the long term outcome in lung function given different allergen exposures, sensitisation, and remodelling status.

Investigating the impact of combined environmental exposures to allergens and viruses in early life is a natural next step because children are not exposed to them in isolation. It may provide insights into how interventions may be best suited to improve overall airway pathology because not all children develop allergen sensitisation, nor do they all develop recurrent wheezing in response to respiratory infections (such as RSV). It has been shown that exposure to certain environmental microbes, such as *Acinetobacter Iwoffii* (*A. Iwoffii*), an environmental bacteria found in cattle farms, provides protection against the airway pathology in asthma when exposed in early life^52^. This brings to light that some environmental exposures may alter the immune response to be protective as opposed to pathogenic and prevent the onset of wheeze and asthma development. It is not clear whether allergen sensitisation primes or mounts a compromised immune response to RSV infection in early life. Further work is needed to clarify the underlying immunological responses and resulting airway pathology upon exposure to multiple environmental agents. The in silico model developed here will lead to further computational investigation of bacterial therapeutic interventions.

A limitation of the in silico model is that we have not explicitly included all innate epithelial cytokines in the model (such as IL-25 and TSLP). However, these cytokines are difficult to quantify reliably, and to date, have not shown a consistent impact in a model of neonatal HDM-induced allergic airways disease^24^. Moreover, some studies show the relevance of IL-33 in childhood asthma^15,37^ and reliability of measurements in a neonatal model. We have therefore focused on IL-33 to generate a more robust in silico model. In future work, the in silico model may be extended to incorporate a more complex multi-scale version describing the specific cytokine interactions of, for example, IL-4, IL-5, and IL-13, involved in allergic sensitisation and airway remodelling, and utilise all existing data for these pathways, rather than grouping cells and cytokines into type 1 and type 2 for simplicity and interpretability.

In summary, our in silico model shows that targeting both the rate of epithelial barrier repair and immune maturation is critical for disease modification in early-onset allergic asthma. Treatments that improve the epithelial barrier repair are being investigated in other diseases, e.g., gut epithelium recovery^53^; however, applications in respiratory medicine are lacking. More experimental work and complementary in silico modelling is needed to understand how environmental exposures impact the development of the early life immune system (influence Th2 skewing) and epithelial barrier repair. Further investigations focused on identifying the specific biomarkers that contribute to slow immune maturation and poor epithelial barrier repair will help distinguish which children may develop allergen sensitisation and airway remodelling that lead to allergic asthma. This may help to identify specific targets for early interventions to enable disease modification (cure) and improve the long-term outcomes of childhood asthma.

## Methods

### Experimental data

All experimental data used in this study is from published results^15–17^. All experimental protocols for animal experiments were previously approved by the appropriate institutional committee (UK Home Office). All methods were carried out in accordance with relevant guidelines and regulations for the previously published studies, and are reported in the original published manuscripts that have been referenced.

The data used was on the measurements of IL-33 (as a marker of epithelial barrier damage), numbers of eosinophils in lung tissue (as a marker of inflammation), and airway resistance in response to methacholine challenge (as a measure of AHR and lung function). Here, we briefly summarise the key points of the protocols (Fig. 2a) that are important for the in silico model structure and development.

Mice were housed under pathogen-free conditions prior to being introduced to HDM at day 3 in neonates^15,16^ and week 6–8 in adults^17^. The mice were challenged with HDM extract intranasally multiple times per week to replicate a constant exposure to HDM. Assessments of immune markers and airway resistance were made weekly throughout the challenges, and 4 weeks post challenges in neonates, and at the end of challenges in adults. Measurements are made at 3 h post challenge^15,16^ and 2 h post challenge^17^. BALB/c mice were used for these experiments^15–17^ as they produce a reliable Th2 inflammatory response that may not be the same in other mice.

We utilised our existing experimental data on IL-33, eosinophilic inflammation, and airway resistance^15,16^ and extracted the remaining data^17^ from published figures using an online tool for data extraction, webplotdigitizer.com^54^. All data was scaled relative to a respective maximum value so that each normalised data set falls in the range [0 1] and all in silico model variables are dimensionless (representing the extent). For comparative purposes, the data from an additional protocol (i.e., where multiple data sets exist for one variable) was translated so that the median value for the control group is consistent across the variable. We used the median of the control group to translate the additional data, instead of the mean of the control group, since the early life lung growth effects skew the mean in the airway resistance. We used the median in the control group across all experimental measures for consistency.

For the validation, we used data on IL-33, eosinophilic inflammation, and airway resistance following corticosteroid^15,37^ and anti-IL-13^15^ treatment in a mouse model. We also used data on IL-33, nasal eosinophil/leukocyte %, and FeNo following Mepolizumab treatment in children^50^. The IL-33 data is presented as a rate ratio (doubling expression) with a 95% confidence interval which we convert to a fold change. Nasal eosinophil/leukocyte % and FeNo data is presented as a mean and standard error in placebo and treatment groups. We extract data on the % predicted forced expiratory volume in 1 s (ppFEV1) following Dupilumab treatment in children^51^. The data is presented as a % mean change from parent study baseline with standard error. In all cases, we calculated the fold change in the treatment vs placebo groups exposed to HDM. All standard errors in the data are propagated to obtain the correct errors in fold change.

### In silico model description

The in silico model of the immune response to HDM exposure in early life describes the rate of change in the extent of epithelial barrier damage (D), eosinophilic inflammation (E), neutrophilic inflammation (N), and airway resistance (A), respectively, in response to HDM exposure (H). The model equations are built using the laws of mass-action for each pathway in Fig. 1c and are described by:

1a $$\frac{{{\text{d}}D}}{{{\text{d}}t}} = \left( {\delta_{H} \left( {1 + \alpha_{H} D} \right)H + \delta_{D} E + \kappa_{R} RD} \right)\left( {1 - D} \right) - \kappa_{B} D,$$

1b $$\frac{{{\text{d}}E}}{{{\text{d}}t}} = \frac{{\kappa^{\prime}_{E} D\left( {1 + \alpha_{I} S} \right) }}{{1 + \alpha_{N} N}} + \kappa_{S} SE - \delta_{E} E,$$

1c $$\frac{{{\text{d}}N}}{{{\text{d}}t}} = \frac{{\kappa_{N} D}}{{1 + \alpha_{E} E}} - \delta_{N} N,$$

1d $$\frac{{{\text{d}}A}}{{{\text{d}}t}} = \kappa_{A} + \kappa_{D} D + \kappa_{F} E + \kappa_{G} N - \delta ^{\prime}_{A} A,$$

where *H* indicates the Heaviside function,

2 $$H = \left\{ {\begin{array}{*{20}l} {0} \hfill & {t < W_{on} ,} \hfill \\ 1 \hfill & {W_{on} \le t < W_{off} {,}} \hfill \\ 0 \hfill & {t \ge W_{off} {,}} \hfill \\ \end{array} } \right.$$

representing the introduction of allergen challenge at week *W*_*on*_ and its removal at week *W*_*off*_. We do not vary the value of *H* to reflect the HDM doses used within the experiments^15–17^ because the HDM dose response is not linear to the immune response and therefore not as simple as changing the value of *H*. Instead, we model a continuous presence of HDM to recreate the desired effects in the experimental protocols.

The remodelling and sensitisation irreversible switches, S and R, are described by:

3 $$S = \left\{ {\begin{array}{*{20}l} 0 \hfill & {E < E_{{{\text{thres}}}} } \hfill \\ 1 \hfill & {E \ge E_{{{\text{thres}}}} , H \ne 0} \hfill \\ \end{array} } \right.$$

4 $$R = \left\{ {\begin{array}{*{20}l} 0 \hfill & {D < D_{{{\text{thres}}}} } \hfill \\ 1 \hfill & {D \ge D_{{{\text{thres}}}} , H \ne 0} \hfill \\ \end{array} } \right.$$

which may only turn on if HDM has been present. We use Heaviside functions for each of the switches as we do not have sufficiently detailed data to constrain the slope if saturating Hill functions were to be used. Using Heaviside functions simplifies the in silico model and increases interpretability.

Th2 skewing in early life is represented by

5 $$\kappa_{E}^{\prime } = \kappa_{E} (1 + \beta e^{ - \lambda t} )$$

where ${\kappa }_{E}$, $\beta$, and λ represent the rate of eosinophil recruitment, extent of Th2 skewing, and rate of immune maturation. The use of an exponential function to describe immune maturation is based on experimental observations demonstrating that eosinophilic inflammation in response to allergen exposure decays exponentially through life to a minimum^55^.

Lung growth in early life is represented by

6 $$\delta_{A}^{\prime } = \frac{{\delta_{A} }}{{1 + \gamma e^{ - \mu t} }},$$

where ${\delta }_{A}$, $\gamma$, and µ represent the rate of resolution in airway resistance, extent of lung growth, and rate of lung growth. The use of an inverse exponential function to describe lung growth is based on experimental observations demonstrating that the baseline airway resistance decays exponentially through life to a minimum^16^. Airway resistance reduces as the airway size increases and we assumed airway size increases with age (to a maximum), implying that airway resistance decreases with age (to a minimum).

We fix the initial conditions for the epithelial barrier damage and eosinophilic inflammation to match the first reading in the respective control groups. Without sufficient time-course data, we assume that the initial condition for the neutrophilic inflammation matches that of the eosinophilic inflammation (note that this does not imply that the absolute initial number of neutrophil cells matches the eosinophils as the variables are relative to their respective maximum values that may differ). Since the baseline airway resistance decreases to a minimum with age (as lung volume increases to a maximum with age), we assume that the maximum airway resistance is the initial condition, and hence after scaling, is set to 1. The parameter values for κ, δ, α, β, $\gamma$, λ, and µ, are all assumed to be real positive constants. Definitions of these parameters and the variables, *D, E, N, A, H, S,* and *R* are provided in Tables 1 and 2, respectively.

**Table 1:** Table of parameters in in silico model (1).

Parameter	Description	Dimension	Value
δ_H_	Rate of epithelial barrier damage caused by protease activity of HDM	Time^−1^	0.0605
α_H_	Proportion of epithelial barrier damage upregulated by a damaged/leaky barrier	–	9.4898
δ_D_	Rate of epithelial barrier damaged caused by eosinophilic inflammation	Time^−1^	0.0745
κ_R_	Rate of remodelling	–	0.1260
κ_B_	Rate of epithelial barrier repair	Time^−1^	0.1069
κ_E_	Rate of eosinophil recruitment	Time^−1^	9.1306
α_I_	Proportion of eosinophil recruitment upregulated by allergic sensitisation	–	0.5690
α_N_	Proportion of Th1 inhibition on Th2	–	166.2375
κ_S_	Rate of additional eosinophilic inflammation due to sensitisation	Time^−1^	0.0361
δ_E_	Rate of eosinophil natural degradation (net loss)	Time^−1^	2.4665
κ_N_	Rate of neutrophil recruitment	Time^−1^	0.0663
α_E_	Proportion of Th2 inhibition on Th1	–	0.0065
δ_N_	Rate of neutrophil natural degradation (net loss)	Time^−1^	0.7909
κ_A_	Rate of increase in airway resistance caused by basal sources such as mucus and airway smooth muscle	Time^−1^	0.1575
κ_D_	Rate of increase in airway resistance due to excessive epithelial barrier damage as epithelial cells are replaced by stiffer extracellular matrix during a dysregulated repair process	Time^−1^	0.1925
κ_F_	Rate of increase in airway resistance due to eosinophilic inflammation, including mucus accumulation, by reducing airflow	Time^−1^	1.2582
κ_G_	Rate of increase in airway resistance due to neutrophilic inflammation, including mucus accumulation, by reducing airflow	Time^−1^	0.0065
δ_A_	Rate of resolution in airway resistance	Time^−1^	2.5245
β	Amplitude of Th2 skewing	–	7.4060
γ	Amplitude of lung growth	–	4.9287
λ	Rate of immune maturation	Time^−1^	0.6982
μ	Rate of lung-growth	Time^−1^	0.7229
D_thres_	Threshold damage for remodelling to occur	–	0.6648
E_thres_	Threshold type 2 cells for sensitisation to occur	–	0.3077
*W*_on_	Week HDM challenge begins	Time	3/7, 7
*W*_off_	Week HDM challenge is removed	Time	3, 6, 8, 10, 12

**Table 2:** Table of variables in in silico model (1).

Variable	Description	Initial value
*D*	Extent of epithelial barrier damage	0.1883
*E*	Extent of eosinophilic inflammation	0.1856
*N*	Extent of neutrophilic inflammation	0.1856
*A*	Extent of airway resistance	1
*H*	Presence of HDM	0
*S*	Sensitisation	0
*R*	Remodelling	0

### In silico model parameterisation

We use a genetic algorithm optimisation to fit in silico model parameters to the normalised experimental data. We begin with an illustrative parameter set that captures the qualitative features of the IL-33, eosinophil cell count, and airway resistance dynamics for the control and HDM cases. Neutrophilic inflammation is treated as a latent variable as we do not have appropriate data to fit to. We then set a search area spanning an order of magnitude around the illustrative parameter set for each of the in silico model parameters with exception for the thresholds D_thres_ and E_thres_, which are bounded by the minimum and maximum of the respective HDM observations, and λ and μ, which are bounded by 0.1 and 1 to avoid blow up/singularities caused by the change in the exponential component. We use a non-weighted cost function that reduces the error between the simulated and observed data. The optimisation is constrained to find parameters that ensure that: the in silico model variables stay within the bounds [0 1]; sensitisation and remodelling occurs by week 4 in neonates exposed to HDM (note we allow the optimisation to determine if remodelling occurs in protocol 1, Fig. 2a, as this is not reported); the baseline airway resistance reduces with age; sensitisation occurs in adult mice exposed to HDM. Following the initial optimisation, we reduce the search area to 2 times above and below the newly optimised set of parameters.

### In silico model validation

To validate the in silico model, we compared simulated and observed results from treatment use. We included both in vivo data from mouse models and clinical data from children (Figure S4) in the validation. Treatments included the common treatment corticosteroid and more recent biologics. We blocked appropriate pathways in the in silico model (representing therapeutic interventions) and quantified the change in the dynamics. For a direct comparison to various data, we calculated the fold change in the treatment vs placebo groups exposed to HDM. Furthermore, we related FEV and FeNo measures (where taken) to AHR with an appropriate scaling. FEV and AHR are inversely related, hence we use the inverse of the fold change in the % change in FEV when comparing our in silico model simulations to clinical data of lung function following Dupilumab treatment. FeNo and AHR increase as lung function decreases, hence we use the fold change in FeNo when comparing our in silico model simulations to clinical data of lung function following Mepolizumab treatment. We assumed that there are no environmental changes in children and assumed that HDM is constant throughout treatment and continues after treatment is removed. We continued the HDM treatment in the mice protocols too to be more realistic.

Corticosteroids are inhaled and may not reach the target destination as effectively as an injected biologic. To reflect the delivery of treatments, we blocked the pathways associated with corticosteroid use by half (0.5x) and the pathways associated with biologics use entirely (0x). The pathways in the in silico model that are blocked in anti-IL-13 use are *dD* and *kA*, reflecting a direct impact on mucus reduction and airway smooth muscle tone. The pathways in the in silico model that are blocked in Mepolizumab use are *kE* and *kS*, reflecting an anti-IL-5 effect with a direct impact on eosinophil recruitment. The pathways in the in silico model that are blocked in corticosteroid use are *dD, kA*, *kE*, and *kS*, reflecting a combined effect of anti-IL-13 and anti-IL-5. The pathways in the in silico model that are blocked in Dupilumab use are *dD, kA*, *aI*, and *kS* reflecting a combined effect of anti-IL-4 and anti-IL-13 with a direct impact on mucus reduction, airway smooth muscle tone, IgE production, and eosinophil recruitment via allergic sensitisation processes. The corresponding pathways for each treatment use are highlighted in Figure S5 in the supplementary.

### In silico model simulation

We used MATLAB version R2022a with built in functions *classdef* (to define the in silico model system), *ode15s* (suitable for stiff equations), and *events* (to create switches under certain conditions) to solve the in silico model under certain conditions. We used the built-in function *ga* (with parallel options) and defined our own cost function to optimise model parameters. We ran the in silico model system to steady state to obtain the long-term behaviour.

## Supplementary Information

Supplementary Information.

## Data Availability

The code that support the findings of this study are available from the corresponding author upon reasonable request.
