# Supplementary material for: Mechanistic modelling of allergen-induced airways disease in early life
Source: Sci Rep. 2025 Jan 2;15:368. doi: 10.1038/s41598-024-83204-x (PMC11696187; doi:10.1038/s41598-024-83204-x)
Supplement: Supplementary file 1 — Supplementary Information. [file 41598_2024_83204_MOESM1_ESM.docx]

**Supplementary Materials**

**Mechanistic modelling of allergen-induced airways disease in early life**

Hannah J Pybus, Prakrati Dangarh, Man Yin Melanie Ng, Clare M Lloyd,

Sejal Saglani, Reiko J Tanaka

**Early-life window of opportunity for successful treatment**

To date, there is no cure for asthma once it is established. To investigate if there exists an early-life window of opportunity for successful treatment use, we used the validated *in silico* model to determine the difference in the treated and untreated immune response to HDM for different durations and initial treatment timings (Fig. S1). Since treatments are generally not used until symptoms are present, we used the validated *in silico* model to simulate treatment use after allergen sensitisation has occurred. The *in silico* model simulation results suggest that the difference in the treated and untreated immune response to HDM differs depending on the duration and timing of the initial treatment, but importantly, the difference also depends on when the comparison between treated and untreated is made.

The *in silico* model simulation results show that across the entire time course there is a significant difference in the treated and untreated airway resistance for early and long treatment use (Fig. S1). However, this difference arises due to the accumulation of treatment use as opposed to the effectiveness of treatment use. At the end of the simulated treatment use we see that an early and short use of treatment is most effective. This may be in part due to the fact that the eosinophilic response to HDM in early life is far greater than in later life (due to Th2 skewing), and therefore an anti-inflammatory treatment appears to have a greater impact. A similar observation is made when considering a timepoint 1 week after simulated treatment use. The *in silico* model simulations show that the current treatments start to lose their effectiveness as the basal airway resistance and eosinophilic inflammatory response to HDM decreases after the first 3 weeks of life. The *in silico* model results suggest that current treatments appear to be most effective whilst both basal airway resistance and eosinophilic inflammatory responses to HDM are high. Crucially, the *in silico* model simulations show that there is no difference in the untreated and treated airway resistance in the long term, for any treatment timing, suggesting that current treatments are not disease modifying (Fig. S1).

The *in silico* model simulation results show that remodelling can be stalled with timely treatment use (Fig. S1). The initial timing of the simulated treatment has a greater impact on when remodelling may occur than the duration of treatment. The *in silico* model results suggest that it is therefore important to use treatments early when trying to prevent remodelling development.


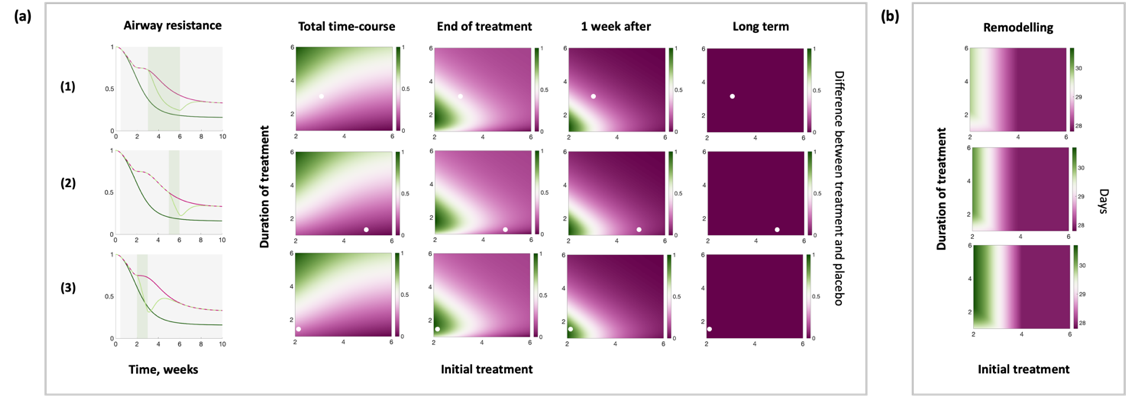


**Fig. S1.** ***In silico* model simulations of treatment use. (a)** Difference between treatment and placebo effects on airway resistance during HDM exposure for various initial starting times and durations of treatment. HDM is introduced at day 3 of life in a nominal virtual mouse and continues throughout the time-course. The first column of plots shows the change in the airway resistance dynamics in response to HDM exposure with treatment use; grey shaded regions represent HDM exposure, light green shaded regions represent treatment use, dark green lines indicate the healthy control (no HDM), pink lines indicate the placebo (HDM and no treatment), and light green lines indicate the treatment (HDM and treatment). The initial time and duration of treatment used in the first column of dynamical plots is shown by the white dots in the same row of plots. **(1)** represents corticosteroid use, **(2)** Mepolizumab, and **(3)** Dupilumab. Total represents the difference between treatment and placebo across the entire time course. End represents the difference between treatment and placebo at the end of the course of treatment. After represents the difference between treatment and placebo 1 week after the end of the course of treatment. Long term represents the difference between treatment and placebo in the long term (week 50 in a mouse). **(b)** The time taken for remodelling to occur once sensitisation has occurred for various treatment regimes.

***In silico* model simulation of an alternative virtual mouse**

Changing the value of the thresholds for the sensitisation and remodelling switch, whilst fixing all other parameters to the optimised values, provides an alternative virtual mouse that captures a different switch order yet retains the key qualitative features of the immune response to HDM (Fig. S2).

**Fig. S2. *In silico* model parameterisation for the alternative virtual mouse.** The immune response dynamics for the alternative virtual mouse parameterisation for each experimental protocol **(1-5)** in Fig. 2a. Shaded regions indicate HDM challenge present. The green lines indicate the *in silico* PBS dynamics and the dark pink lines the *in silico* HDM dynamics. The dotted lines in the epithelial damage and eosinophilic inflammation plots indicate the thresholds for the switches. The resulting switch states are shown by the colours of the bars above the plots, where pink indicates remodelling, and orange indicates sensitisation.

**Immune maturation rate impacts long term lung function**

To investigate the impact of developmental changes of children on the immune response to HDM and resulting long term lung function, we evaluated the simulated long-term eosinophilic inflammation and lung function for virtual mice with different rates of immune maturation and lung growth (Fig. S3). The *in silico* model simulations show that the rate of immune maturation has a greater impact on the eosinophilic inflammation and airway resistance than lung growth since there is little to no change in the results when varying lung growth (Fig. S3). Slow rates of immune maturation increase the eosinophilic inflammatory response to HDM in early life and are detrimental to lung function shown by the increase in airway resistance (Fig. S3). Fast rates of immune maturation decrease the eosinophilic inflammatory response to HDM in early life and are beneficial to lung function shown by the decrease in airway resistance. The time taken for remodelling and sensitisation to occur increases as the rate of immune maturation increases; for fast rates of immune maturation, sensitisation may not occur (Fig. S3).

**Fig. S3. *In silico* model simulations of the immune response to HDM with changes in lung growth and immune maturation.** **(a)** Total eosinophilic inflammation and airway resistance in response to HDM relative to controls, for different rates of immune maturation and lung growth. High values indicate fast immune maturation and lung growth, whilst low values indicate slow immune maturation and lung growth. The rate of immune maturation decreases from the optimised value in the direction of the black arrows and increases from the optimised value in the direction of the grey arrows for a fixed value of lung growth. Green represents an improvement in lung inflammation and function, whereas pink represents impairment. **(b)** The time taken for remodelling and sensitisation to occur; for high values of immune maturation, sensitisation may not occur (shown by white region).

***In silico* model simulation of current treatment strategies in children**

We validated the parameterised *in silico* model by confirming that the *in silico* model simulations can capture the main features of the experimental observations reported in published studies for two treatment strategies using biologics given after the onset of disease in children (Fig. S4). Biologics are modelled to entirely block their targeted pathways since they are injected. The *in silico* model simulations are in agreement with the published studies showing that: there is a reduction in inflammation with no change in damage and varied outcomes in AHR following Mepolizumab treatment^43^, and there is an improvement in lung function following Dupilumab treatment^44^.

The outcomes in Mepolizumab treatment use are varied; Mepolizumab is more effective in eosinophilic high patients and not those with remodelling. There is a slight quantitative discrepancy between the *in silico* model simulations and the clinical data for the change in AHR (lung function) following Mepolizumab treatment. The discrepancy may be due to the use of a different lung function measurement, *i.e.*, FeNo as opposed to airway resistance. Alternatively, it may be due to the parameterisation of the *in silico* model, where the eosinophilic inflammation is a large contributor to airway resistance (through optimised parameter values) and hence treatments reducing eosinophilic inflammation like Mepolizumab greatly impact airway resistance in the *in silico* model. Further calibration using data from children, instead of mice, will help to advance the *in silico* model to simulate different environmental exposures and treatment regimes in humans.

**Fig. S4.** ***In silico* model simulations of current treatment strategies.** Validation of the parameterised *in silico* model against current treatment strategies. All data points (circles with 95% confidence interval bars or SEM) and simulation dynamics (solid lines) are plotted as a fold change in treatment application vs placebo with HDM present. The treatment regimens tested are **(1)** Mepolizumab applied to children^43^, and **(2)** Dupilumab applied to children^44^, after the disease onset.

**Fig. S5.** **Pathways blocked in the *in silico* model simulations of current treatment strategies.** The pathways in the *in silico* model that are blocked in **(a)** anti-IL-13, **(b)** Mepolizumab, **(c)** corticosteroid, and **(d)** Dupilumab use are shown above in light grey. Diagrams created in BioRender (2024) <https://biorender.com/l66k589>.

***In silico* model simulation of different phenotypes**

We conducted a sensitivity analysis of the model parameters to highlight which pathways influence the long-term lung function. The LHS-PRCC sensitivity analysis demonstrates that the most influential parameters on the long-term airway resistance (as a measure of lung function and an output of the model) are those that appear within the airway resistance equation (Fig. S6a). The rate of epithelial barrier repair (κ_B_) and immune maturation (λ) are significant parameters (p-value < 0.05) and contribute to the long-term change in airway resistance. The effects of changes in epithelial barrier repair and immune maturation on the long-term airway resistance have been investigated in the main results (Fig. 5 and 6).

The robustness of our main results (Fig. 5 and 6) to parameter variation were confirmed by calculating the minimum duration of HDM exposure required to establish the allergic asthmatic state (remodelling and sensitisation occur) and allergic state (sensitisation occurs) for 5000 different parameter sets that varied by 50% from the nominal values, representing a variety of phenotypes. The minimum duration is longer for faster barrier repair and faster immune maturation in all biologically relevant parameter variations (Fig. S6b-c).

**Fig. S6. Sensitivity analysis of the *in silico* model parameters. (a)** LHS-PRCC for the long-term airway resistance in response to HDM. 1000 parameter sets varied by an order of magnitude. The parameter sensitivities are in ascending order and follow the same colour coding as that in Fig. 1 to show which terms correspond to which equation. Those with stars indicate parameters with statistically significant PRCC values (p-value < 0.05) that influence the long-term airway resistance. **(b)** The minimum duration of HDM exposure (T_min_) required to establish the allergic asthmatic state (remodelling and sensitisation occur) for fast/slow barrier repair and **(c)** to establish the allergic state (sensitisation occurs) for fast/slow immune maturation for 5000 different parameter sets varying by 50% from the nominal values. Colour coding for the allergic and allergic asthmatic states match those in Fig. 5 and 6 (and throughout).
